# 

**Published:** 1842-07

**Authors:** 


### CONTENTS

### OF NO. I.

### ORIGINAL COMMUNICATIONS.

### ESSAYS AND CASES.

ART. I.—A History of the Improvements which Practical Medicine has derived from Auscultation; being the Essay to which the Medical Society of Bordeaux awarded its prize in November, 1839. By G. PEYRAUD, Doctor of Medicine of the Faculty of Paris, Corresponding Member of the Bordeaux Medical Society. *Quæ fundata sunt in natura, crescunt et perficiuntur; quæ vero in opinione, variantur, non augentur.*—Baglivi. Translated from the French for the Western Journal of Medicine and Surgery, by CHARLES A. POPE, M. D., of St. Louis, Lecturer on Anatomy and Surgery, Physician to the St. Louis Dispensary, &c., &c. 1

### BIBLIOGRAPHICAL NOTICES.

ART. II.—The New England Quarterly Journal of Medicine and Surgery. Vol. I, No. 1, for July, 1842. Boston, pp. 156. - - - - - - - 59

ART. III.—The American Journal and Library of Dental Science. Vol. II, No. 4, June, 1842. Baltimore. 61

### SELECTIONS FROM AMERICAN AND FOREIGN JOURNALS.

Secondary hemorrhage after amputations—comparative advantages of circular and flap amputations. - - 63

Fracture of the tibia, with avulsion of the internal malleolus, and penetrating wound of the ankle-joint; consecutive abscesses; necrosis of the tibia, and resection of its inferior extremity—recovery. . . . . . . 65
Comparative frequency of tuberculous disease. . . 67
Diabetes mellitus. . . . . . . 69

ORIGINAL INTELLIGENCE.

Experiments on Mesmerism. . . . . . 71
College of Professional Teachers. . . . 79
Influence of Auscultation on Practical Medicine. . 80
Correction. . . . . . . . 80

